# Effect of a Fixed Combination of Curcumin, Artemisia, Bromelain, and Black Pepper Oral Administration on Optical Coherence Tomography Angiography Indices in Patients with Diabetic Macular Edema

**DOI:** 10.3390/nu14071520

**Published:** 2022-04-06

**Authors:** Flavia Chiosi, Michele Rinaldi, Giuseppe Campagna, Gianluigi Manzi, Vincenzo De Angelis, Francesco Calabrò, Luca D’Andrea, Fausto Tranfa, Ciro Costagliola

**Affiliations:** 1 Department of Ophthalmology, Monaldi Hospital, Azienda Ospedaliera dei Colli Via Leonardo Bianchi, 8013 Napoli, Italy; gianluigi.manzi@ospedalideicolli.it; 2 Department of Ophthalmology, University of Campania “Luigi Vanvitelli”, 80100 Napoli, Italy; michrinaldi@libero.it; 3 Department of Medical-Surgical Sciences and Translational Medicine, University of Rome “La Sapienza”, 00161 Roma, Italy; gius.campagna@gmail.com; 4 Eye Clinic, Azienda Ospedaliera A. Cardarelli, 80131 Naples, Italy; vincedeangelis2008@alice.it (V.D.A.); francescocalabro@hotmail.it (F.C.); 5 Eye Clinic, Department of Neuroscience, Reproductive Sciences and Dentistry, University of Napoli “Federico II”, 80138 Napoli, Italy; lucadandrea@gmail.com (L.D.); fausto.tranfa@unina.it (F.T.); ciro.costagliola@unimol.it (C.C.)

**Keywords:** diabetic retinopathy, diabetic macular edema, curcumin, artemisia, bromelain, black pepper, OCTA, vascular flow density

## Abstract

Background: To investigate the effects of a fixed combination of Curcumin (200 mg), Artemisia (80 mg), Bromelain (80 mg), and Black pepper (2 mg) on vascular parameters in mild to moderate diabetic macular edema (DME). Design: Prospective, case-control study. Methods: Fifty-six patients affected by diabetes mellitus type II were enrolled in the study. Twenty-eight patients with DME received 2 tablets/day, before meals of a dietary complementary supplement containing in fixed combination Curcumin (200 mg), Artemisia (80 mg), Bromelain (80 mg), and Black pepper (2 mg) (Intravit^®^, OFFHEALTH Spa, Firenze, Italy) for 6 months. Twenty-eight age-matched subjects affected by diabetes mellitus type II were given placebo and served as control group. Patients underwent best correct visual acuity (BCVA), swept optical coherence tomography (OCT), and OCT-Angiography (OCTA). OCTA images of the superficial capillary plexus (SCP) and deep capillary plexus (DCP) were obtained for each eye. By the end of the follow-up patients were defined responder to the therapy when a decrease of more than 30 μm was registered in central retinal thickness (CRT) measurement, while a poor responder was determined by the absence of reduction or an increase in central retinal thickness at 6 months. We assessed the foveal avascular zone (FAZ) area, vessel density and quantified the number of microaneurysms in each layer. Results: A significant improvement of BCVA and CRT reduction was recorded at 6 months follow-up in the dietary complementary supplementation group compared to control (respectively *p* = 0.028 and *p* = 0.0003). VD of the total capillary plexus, microaneurysms count, glycaemia and HbA1c did not vary over the follow-up period between groups. Within the Intravit^®^ group, poor responders tended to show a larger FAZ area, more microaneurysms, and a lower VD in the DCP compared to the good responders group (*p* < 0.0001). Conclusions: A fixed combination of Curcumin, Artemisia, Bromelain, and Black pepper oral administration may have a positive impact on central retinal thickness, visual acuity, and VD of the DCP in compensated type 2 diabetic patients with mild DME.

## 1. Introduction

Diabetic macular edema (DME) is a clinical condition characterized by the storage of exudative fluid in the macula, the most important area of the retina accountable for central vision. Clinically significant macular edema can be present at all stages of diabetic retinopathy (DR) and is positively correlated with the duration of diabetes, affecting about 30% of diabetic patients during 25–30 years of disease [1].

Early detection of DR through screening programs and timely laser treatment have improved the systemic management of diabetes in developed countries, making DME more frequent than proliferative DR among people with diabetes [2]. When plasmatic glucose levels become high in a chronic trend, which is typical of diabetes, such condition initiates a number of biochemical reactions leading to ischemia and, lately, to an hyper-production of different growth factors. Within these, vascular endothelial growth factor (VEGF) has a predominant role, promoting both growth and increased permeability of endothelial cells [3,4,5].

Inflammation represents one of the main pathological features of DME. Increased inflammatory mediators and pro-inflammatory cytokines induce a persistent low-grade inflammation in the diabetic retina, resulting in leucstasis, leucocyte activation, and modification of the sealing function of the blood–retinal barrier. Thoroughly, this condition suggests that ocular diabetic complications and inflammation are connected, and that the levels of circulating inflammatory agents may prognosticate the outbreak and progression of diabetic retinopathy [6,7]. DME is the consequence of microvascular alterations induced by both vascular and inflammatory mechanisms, which lead to the disruption of the blood–retinal barrier.

Based on the severity of DME, several therapies are available to treat such condition.

Although in the current pathway for DME, there is no consensus in terms of estimating therapy response, especially in the early phases of the DME. Definitions of response to therapies are still not uniformly agreed upon. The National Institute for Health and Care Excellence (NICE) determined OCT parameters comprising central retinal thickness (CRT) in developing guidelines for pharmacological therapies for DME. (http://www.nice.org.uk/guidance/ta346e274 accessed on 1 October 2018 )

NICE and the National Health Service (NHS) recommends pharmacological treatments for DME in eyes with CRT > 400 μm. Such therapies include the utilization of intravitreal injections of antivascular endothelial growth factor (anti-VEGF).

Guidelines for the management of DME inferior to 400 microns are still under investigation.

In traditional medicine, some natural molecules, like Curcumin, Artemisia, Bromelain, and Black Pepper, are able to decelerate the advance of diabetes complications. Antioxidant, anti-inflammatory, and anti-proliferative properties have been associated with the use of curcumin demonstrating beneficial effects on multiple disorders. Efficacy in limiting the pathophysiological changes in diabetes has been shown in several animal studies [8]. Artemisia restore islet ß-cell function and attenuates insulin resistance in diabetes, and seems to exert potential effects also on diabetic complications, i.e., diabetic kidney disease, diabetic retinopathy, diabetic cardiovascular disease, and cognitive impairment [9]. Studies on bromelain, a cysteine protease from pineapple juice and stems, proved an efficiency similar to that of standard anti-inflammatory drugs, even though its mode of action remains unclear. Bromelain enhances immune defenses and inhibits angiogenesis; moreover, due to its anti-angiogenic and anti-inflammatory activities, it may be helpful in different therapeutic areas [10,11]. Black pepper is a significant healthy food with antioxidant properties, anti-microbial potential, and gastro-protective characteristics. One of the main property lies in the free-radical scavenging activity of black pepper and its active components might be useful in chemoprevention and regulation of the progression of the tumor growth. Furthermore, the key alkaloid components of Piper Nigrum, that is piperine, facilitates cognitive brain functioning, boosts nutrient absorption, and improves gastrointestinal functionality [12].

Recently the imaging of the ocular circulation can be displayed through optical coherence tomography angiography (OCTA). OCTA gives an exhaustive examination of the vascular layers along with significant functional information on the blood reservoir of the retina and choroid, using the variation signals generated by moving red blood cells and other blood components. [13].

OCT-A pictures can be subjectively evaluated for the presence of pathology or post processed to create quantitative data that can be assessed objectively. Multiple studies applied perfusion indices such as vessel density (VD) and flow index to quantitatively analyze OCT-A images.

Recently, a set combination of the above-cited molecules became available. The combination was fixed based on daily nutritional requirements, aiming for a synergistic effect between the antioxidant, anti-inflammatory, and anti-proliferative properties of each component. No interaction with antidiabetic drugs has been reported for any of the molecules included in the fixed combination [14]. The purpose of this preliminary study is to evaluate the effects of a fixed combination of Curcumin (200 mg), Artemisinin (80 mg), Bromelain (80 mg), and Black pepper (2 mg) on visual acuity, vessel density of the retinal microvasculature, and central retinal thickness in patients affected by DME.

## 2. Patients and Methods

This prospective case control study comprises 56 patients with type 2 diabetes with an established diagnosis of DR and subjected to an extensive ophthalmologic evaluation including swept-source optical coherence tomography (SS-OCT) and OCT-Angiography (OCTA), in the Department of Ophthalmology at AORN dei Colli and Cardarelli Hospital, Napoli, Italy. Informed consent was received from each patient before beginning the study, and a detailed description of the procedures to be used and the aim the work was provided. The study was registered at clinicaltrial.gov (registration number: NCT04742829).

The degree of DR severity was defined in each patient using the International Clinical Diabetic Retinopathy and diabetic Macula Edema Severity scale.

Inclusion criteria are as follows: (a) women/men aged between 40 and 70 years; (b) compensated type 2 diabetic patients (both genders) under therapy with anti-hyperglycemic drugs (oral and/or subcutaneous); (c) diabetes duration for at least 5 years and not exceeding 15 years; (d) non-proliferative diabetic retinopathy, limited to the presence of some microaneurysms; (e) established DM diagnosis according to the American Diabetes Association criteria; (f) best corrected visual acuity (BCVA) ≥ 20/50 EDTRS charts; (g) absence of cataract; (h) absence of systemic complications of diabetes. Conversely, we excluded: (a) uncompensated diabetes; (b) patients with the diagnosis of proliferative retinopathy; (c) pregnancy, breast-feeding; (d) history of intolerance to the study medications; (e) diseases of the macula (e.g., foveal scar, foveal atrophy, etc.,) or other reasons of macular edema (e.g., exudative macular degeneration, uveitis, ocular inflammation, epiretinal membrane, etc.); (f) previous ocular surgery.

Central retinal thickness (CRT) lower than 400 μm was considered as mild DME according to the National Institute for Health and Care Excellence guidelines (http://www.nice.org.uk/guidance/ta346e274 accessed on 1 October 2018).

Patients were separated into two groups: the Intravit group and the control group.

The Intravit group comprised twenty-eight patients receiving 2 tablets/day, before meals of a dietary complementary supplement containing in fixed combination of Curcumin (200 mg), Artemisinin (80 mg), Brome-lain (80 mg), and Black pepper (2 mg) (Intravit^®^, OFFHEALTH Spa, Firenze, Italy) for 6 months. The other twenty-eight patients served as control group.

At baseline, and after 3 and 6 months, the following examinations were performed: BCVA, anterior segment slit-lamp and fundus examination, measurement of central retina thickness (CRT), micro-aneurysms (Mas) quantification, fundus photography, foveal avascular zone (FAZ), and OCT/OCT-A scans were recorded using the swept source Topcon DRI OCT Triton (Topcon Corporation, Tokyo, Japan).

Within the Intravit group, a sub-analysis was performed on the basis of the patient’s response to therapy. A good responder was determined by a reduction of more than 30 μm in central retinal thickness and a poor responder was described by unaltered or increased central retinal thickness compared to baseline. OCTA parameters (VD and FAZ) were compared between these two groups.

### 2.1. Scanning Protocol and Quantitative Analysis of OCTA Images

One eye of each patient was chosen for the analysis. Both eyes of each patient were scanned, then the eye with higher quality scans was considered. The acquisition protocol consisted of a macular line and 6 × 6 mm macular cube on OCT and OCT-A. All scans were centered on the fovea, and the superficial capillary plexus (SCPs) and deep capillary plexus (DCP) were identified using the IMAGENET 6.0 automated layer detection tool. The VD was extracted from the retinal nerve fiber layer and ganglion cell layer for the SCPs calculation and from the inner plexiform layer and inner nuclear layer for the DCPs. 3D datasets were analyzed to determine retinal thickness. To quantify the OCTA slabs, we evaluated the total vascular flow density, FAZ area and quantified the number of microaneurysms in each plexus. Fusiform dilation two-fold thicker than the capillary diameter and saccular capillary ends were described as microaneurysms in this study. Two retinal specialists (C.F. and D.V.) manually counted the number of microaneurysms in a 6 × 6-mm area of each vascular layer. The FAZ area was determined as an avascular central area, and the edge of the FAZ was manually outlined by two retinal specialists (F.M.E. and R.M.).

### 2.2. Sample Size

To calculate the sample size, we conducted a pilot study where we considered the values at baseline and the end of the study of Central Retinal Thickness in Intravit group and control group. Using the procedure *proc ttest* for independent groups, we estimated the pooled standard deviation *s* = ±54.14. We hypothesized as differences clinically plausible d = 65 μm. To estimate the sample size needed to conduct the study, we applied the *proc power twosamplemeans test = diff* with α = 0.01 and power = 90%. The result of this procedure indicated a total sample size n = 46 namely n = 23 for Intravit group and n = 23 for control group.

Taking into account a drop-out of 20%, the number of subjects of both groups was about n = 28 and therefore the study was conducted on 56 patients. The calculation of sample size was performed by SAS v. 9.4 (SAS Institute Inc., Cary, NC, USA).

### 2.3. Statistical Analysis

Continuous variables were showed as mean ± standard deviation (SD), 95%CI (Confidence Interval) or median, 95%CI and (minimum and maximum). The regularity of the continuous variables and of the residuals was tested by Shapiro–Wilk test and checking the Q-Q plot. The categorical variable gender was compared to Intravit vs. control group using the X^2^ test and presented as absolute frequencies and percentages. *t* Student test was used to compare the age, superficial capillary plexus, deep capillary plexus, and foveal avascular zone between Intravit vs. control group. Mann–Whitney test was performed to compare visual acuity at baseline between Intravit vs. control group. Longitudinal data analysis of central retinal thickness (CRT), glycemia, glycosylated hemoglobin (HbA1c), microaneurysm, and total vascular flow density (TVFD) was performed using a generalized linear mixed model method (GLIMMIX) with repeated measures. This method allowed to fix the distribution of data: with continuous variables the distribution was Gaussian; while for the microaneurysm the distribution was Poisson. The Poisson function was chosen due to the fact that microaneurysms were considered as a counting variable. Poisson function was applicable because there was no overdispersion. To analyze the overall treatment effect, ($Y_{t}=\beta_{0}+\beta_{1}X+\beta_{2}Y_{t0}$) and treatment effects at two follow-up measurements (three and six months) ($Y_{t}=\beta_{0}+\beta_{1}X+\beta_{2}Y_{t0}+\beta_{3}time+\beta_{4}X*time$) where $Y_{t}$ = the outcome measured at three and six months, *X* = treatment group, $\beta_{1}$ = overall treatment effect, $\beta_{2}$ = regression coefficient relative at baseline value of the outcome variable, $\beta_{3\;}$ and $\beta_{4}$ = regression coefficients for the different follow-up measurements and $Y_{t0}$ = outcome variable measured at baseline we applied the longitudinal analysis of covariance performed by GLIMMIX with baseline values considered as covariate, namely with adjustment for the baseline value of the outcome variable. The distribution follows the rule previous. In general, in an RCT the adjustment for the baseline value of the outcome variable is always advised to estimate a treatment effect order to avoid overestimation of treatment effect [15]. The homoscedasticity was verified by checking studentized residuals vs. fitted values plot. The Tukey method was used to correct multiple comparisons. A *p* < 0.05 was considered statistically detectable. All statistical analyses were carried out using SAS v. 9.4 (SAS Institute Inc., Cary, NC, USA).

## 3. Results

Fifty-six patients were included in this study and subdivided in Inravit and control group. The mean age was matched between the two groups: (Intravit group: 71.11 ± 5.75; 95% confidence interval: (95%CI: 68.88 to 73.34 years) and control group: 71.04 ± 5.63; (95%CI: 68.85 to 73.22 years), *p* = 0.96).

In the Intravit group, there were 17 (51.52%) males and 11 (47.83%) females, while in control group there were 16 (48.48%) males and 12 (52.17%) females. There was no association between sex and groups (*p* = 0.79).

Visual acuity at baseline had not shown any difference between groups: (Intravit group: 0.40; (95%CI: 0.32 to 0.40) and (min to max) (0.25 to 0.40); and control group: 0.32; (95%CI: 0.32 to 0.40) and (min to max) (0.20 to 0.40), *p* = 0.29). These values converted into distance correspond to: (Intravit group: 20/50, (95%CI: 20/63 to 20/50) and (min to max) (20/80 to 20/50); and control group: 20/63, (95%CI: 20/63 to 20/50) and (min to max) (20/100 to 20/50).

**Figure 1:**
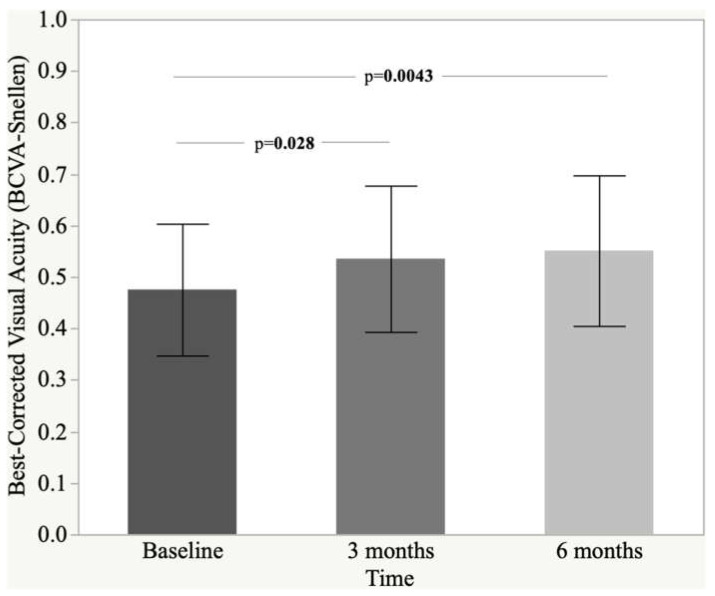
Histogram and standard deviation of best-corrected visual acuity (BCVA-Snellen).

Figure 1 shows BCVA changes over the follow-up period (baseline, month 3 and month 6, *p* = 0.0037). A significant improvement of BCVA was recorded already after 3 months of a dietary complementary supplementation of Intravit^®^ (baseline 0.47 ± 0.13 vs. three months 0.53 ± 0.14, *p* = 0.028). Three months later (after 6 months of follow-up), a further progress of BCVA was seen (0.55 ± 0.15, *p* = 0.0043 vs. baseline). The difference between three and six month checks was not significant (*p* = 0.77) (Figure 1).

In Table 1 were showed the results of the follow-up relatively to variables central retinal thickness (CRT), glycemia, glycosylated hemoglobin (HbA1c), microaneurysm, and total vascular flow density (TVFD) between placebo and Intravit group.

In the control group, differences were evidenced in the three temporal points for none of the variables analyzed. In the Intravit group, only CRT and TVFD showed a difference in the follow-up (both, *p* < 0.0001). Post-hoc analysis highlighted the difference of the means of CRT between baseline versus three and six months (*p* = 0.0008 and *p* < 0.0001, respectively) and in the means of TVFD between baseline versus three and six months (*p* = 0.015 and *p* < 0.0001, respectively) and three months versus six months (*p* = 0.002). CRT and TVFD have an opposite trend; in fact, CRT decreases over time with values quite different in the follow-up, while FVD increases by about one percentage point in the three temporal points analyzed.

Finally, CRT reduction was observed in 10.71% of the control group population, while in the Intravit group 71.43% of the patients showed a CRT decreasing at the end of follow-up, therefore comparing the two proportions there is an evincible difference and equal to *p* < 0.0001.

Table 2 contains the estimates of the overall treatment effects and the estimates of the therapy effects individually for the two follow-up measurements of the variables previously described. 

The overall treatment effects was negative for CRT, glycemia, and HbA1C with values of −24.24 μm, −6.81 mg/dL, and −0.07%; so, on average over time the Intravit group has −24.24 μm, −6.81 mg/dL, and −0.07% lower CRT, glycemia, and HbA1C compared to the control group. These comparisons were all significantly detectable (*p* = 0.016, *p* = 0.04 and *p* = 0.02, respectively).

Total vascular flow density showed a positive value of overall treatment effect (2.15%, *p* < 0.0001) indicating that TVFD was higher in Intravit group with respect to the control group.

At the two follow-up evaluations, the longitudinal analysis revealed effects of −19.74 μm (*p* = 0.008) for CRT and 0.07% (*p* = 0.0005) for TVFD at three months indicating that to this temporal point the values of CRT were lower in Intravit group with respect to control group; while the value of TVFD was higher in Intravit group when compared to the control group.

At six months all comparisons (Intravit vs. control) were statistically detectable (−28.89 μm, *p* = 0.0003; −7.63 mg/dL, *p* = 0.02; −0.09%, *p* = 0.017; −0.09% which corresponds to −6.76%, *p* = 0.026 and 0.10%, *p* < 0.0001). The signs of the estimates at this time point were in line with those of the overall treatment effects and the meaning was the same as described before.

Table 3 shows superficial capillary plexus (SCP), deep capillary plexus (DCP), and foveal avascular zone (FAZ) between good and poor responders.

**Figure 2:**
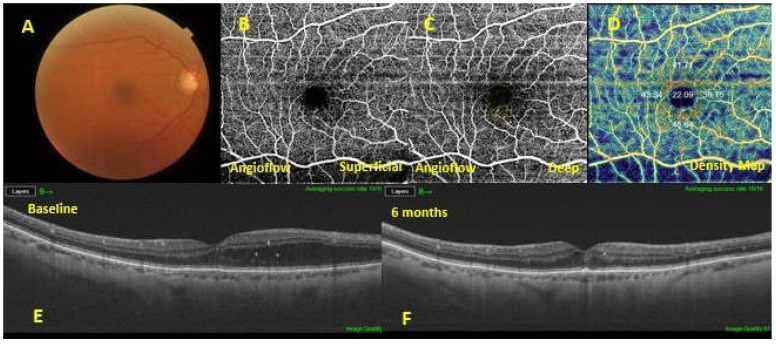
A diabetic macular edema (DME) eye exhibiting a good response to dietary oral supplementation. Baseline fundus pho-tography (**A**). Optical coherence tomography angiography showing absence of microaneurysms in the superficial capillary plexus (**B**) , sporadic microaneurysms in the deep capillary plexus (**C**) and increased vascular flow density (**D**). The 6-months follow-up swept-source optical coherence tomography (SS OCT) (**F**) showed a resolution of the macular edema, compared with the baseline SS OCT (**E**).

**Figure 3:**
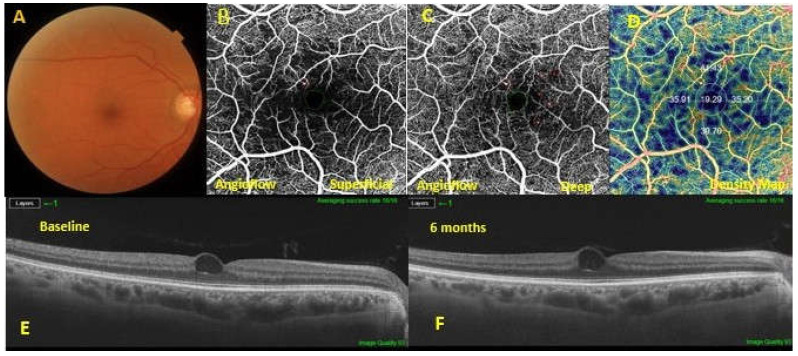
A diabetic macular edema (DME) eye exhibiting a poor response to dietary oral supplementation. Baseline fundus photography (**A**). The optical coherence tomography angiography showed microaneurysms in both the superficial capillary plexus (SCP) (**B**) and the deep capillary plexus (DCP) (**C**). At the level of the DCP, a lower vascular flow density was evidenced in the large foveal avascular area (**D**). In the 6-months follow-up swept-source optical coherence tomography SS OCT (**F**), macular edema was not improved compared to the baseline SS OCT (**E**).

Both DCP and FAZ showed a difference statistically detectable (*p* < 0.0001 and *p* = 0.0013, respectively); in fact, between good responders vs. poor responders the vascular flow density of DCP resulted higher in the good group; while the area of the FAZ resulted higher in the poor group (Figure 2 and Figure 3). No differences were detected at the level of the SCP between the two groups.

## 4. Discussion

Preliminary results from this study demonstrated that 6 months of a dietary complementary supplement containing in fixed combination, Curcumin (200 mg), Artemisia (80 mg), Bromelain (80 mg), and Black pepper (2 mg) (Intravit^®^, OFFHEALTH Spa, Firenze, Italy), additionally to the present therapy for the treatment of diabetes, improves CRT, BCVA, and VD in patients with mild DME.

Although diabetic patients undergo more often ophthalmic evaluations than the average population, DME affects 1 in 3 people with diabetes and nearly 27% of patients present signs of macular edema within 9 years of diabetes outbreak [16,17].

Individually, Curcumin, Artemisinin, Bromelain, and Black pepper exert various therapeutic effects useful for retinal diseases [18,19]. Experimental studies have demonstrated that intravitreally injected artemisia reverses iris and retinal neovascularization, macular edema, vascular dilatation, and tortuosity and fluorescein leakage in rabbits and monkeys models [20]. These evidence suggest that artemisia has great capability in the therapy of DR as an antiangiogenic drug, regulating mitochondrial function and balancing oxidative stress [9]. From the in vivo and in vitro data that are currently available, bromelain demonstrates anti-inflammatory effects, similar to those exerted by non-steroidal anti-inflammatory drugs, reducing plasma fibrinogen levels, playing on the fibrinolytic activity, and reducing bradykinin levels [21]. These mechanisms of action have a particular influence on edema formation.

Black pepper and piperine have been found to promote and modulate the elevated levels of inflammatory biomarkers, such as IL-1β, TNF-α, and NF-κB, and malondialdehydes (MDA) in diabetic mice models [22,23,24].

In our study a significant resolution of the DME was observed in 71.4% of the treated patients. Therefore, a sub-analysis in the Intravit group to investigate the OCTA indices of the SCP and DCP and its association with the response to the oral supplement therapy was conducted.

First, a comparison of the OCTA findings between DME eyes that well responded to Intravit supplementation and those that poorly responded to the same therapy was performed. The mean area of the FAZ was significantly higher in the poor responder compared to the good responder; whereas in the good responder, an increase of the VD in the DC occurred. Moreover, poor responder DME eyes showed more microaneurysms, lower vascular flow density in the DCP, and a larger FAZ area, although no significant differences were evidenced between the two groups in the vascular flow density of the SCP (Table 3). The disparity in the DCP parameters between the two groups imply that the wholeness of the perifoveal DCP, but not that of the SCP, is related with the therapy response to Intravit agents.

In this regard, screening of such parameters in baseline examinations might help to give a prognostic evaluation of the treatment response.

The mechanism inherent in the correlation between the low-flow density in the DCP and treatment resistance to Intravit agents remains to be clarified. One plausible reason is that the VEGF expression in the ischemic deep retina in the deficiency of the DCP weakens the efficacy of such agents. The DCP might be involved in the removal of excess fluid from the retina and consequently its impairment might predispose to the localized storage of fluid and its consequent resistance to fluid uptake.

Other studies provided also evidence of the efficacy of oral dietary supplements in the treatment of diabetic retinopathy [25].

In the past, carotenoids have been widely investigated for their neuroprotective and anti-inflammatory role in the management of diabetic retinopathy [26]. Functional parameters such as multifocal electroretinography have been used to estimate the efficacy of carotenoids supplementation in patients affected by DME [27]. Certainly, oral supplementation with natural agents holds huge advantage of being a non-invasive therapy with apparently no damaging effects. The current issue is to determine whether it is an effective therapy for diabetic retinopathy and to identify which agent would be most appropriate to treat these individuals.

To the best of our knowledge, no previous studies have evidenced the effects of a fixed combination of Curcumin (200 mg), Artemisia (80 mg), Bromelain (80 mg), and Black pepper (2 mg) on CRT, BCVA, and VD in patients with DME.

The limitations of the present study include the short-term follow-up and the fact that we cannot determine which component is accountable for the improvement seen in CRT, BCVA, and VD. Further studies focused on the effects of the individual components are warranted.

## 5. Conclusions

In conclusion, data from this preliminary study strongly recommend the positive impact of this treatment on mild DME in compensated type 2 diabetic patients with non-proliferative diabetic retinopathy. The use of this association may be helpful to strengthen conventional therapy for DR and to treat the beginning of complications, such as DME, and may represent a potential option in the armamentarium of ophthalmologists for the treatment of these patients.

## Figures and Tables

**Table 1:** Descriptive information of the parameters in control and intravit groups according to temporal points.

Parameter	Control			*p*	Intravit			*p*
	Baseline Mean ± SD (95%CI)	Three Months Mean ± SD (95%CI)	Six Months Mean ± SD (95%CI)		Baseline Mean ± SD (95%CI)	Three Months Mean ± SD (95%CI)	Six Months Mean ± SD (95%CI)	
CRT (μm)	299.32 ± 53.05 (278.75 to 319.89)	299.21 ± 52.55 (278.84 to 319.59)	300.82 ± 53.57 (280.05 to 321.60)	0.09	325.89 ± 70.66 (298.49 to 353.29)	297.96 ± 48.86 (279.02 to 316.91)	290.43 ± 48.22 (271.73 to 309.13)	<0.0001
Glycemia (mg/dL)	170.54 ± 28.43 (159.51 to 181.56)	171.07 ± 28.39 (160.06 to 182.08)	172.14 ± 26.92 (161.70 to 182.58)	0.08	168.00 ± 29.87 (156.42 to 179.58)	163.00 ± 25.63 (153.06 to 172.94)	162.46 ± 25.85 (152.44 to 172.49)	0.12
HbA1C (%)	7.14 ± 0.69 (6.88 to 7.41)	7.15 ± 0.68 (6.89 to 7.42)	7.19 ± 0.67 (6.93 to 7.45)	0.09	7.12 ± 0.71 (6.84 to 7.40)	7.09 ± 0.68 (6.83 to 7.35)	7.08 ± 0.67 (6.82 to 7.34)	0.15
Microaneurysm (count)	3.86 ± 1.76 (3.17 to 4.54)	3.86 ± 1.76 (3.17 to 4.54)	3.86 ± 1.76 (3.17 to 4.54)	1.00	4.18 ± 1.94 (3.42 to 4.93)	4.07 ± 1.90 (3.33 to 4.81)	3.89 ± 1.89 (3.16 to 4.63)	0.87
TVFD (%)	24.04 ± 1.53 (23.45 to 24.64)	23.46 ± 1.93 (22.71 to 24.21)	23.89 ± 1.89 (23.16 to 24.62)	0.15	24.12 ± 2.19 (23.27 to 24.97)	25.21 ± 2.19 (24.36 to 26.05)	26.54 ± 1.56 (25.93 to 27.14)	<0.0001

Abbreviation–CRT: central retinal thickness; TVFD: total vascular flow density. Post-hoc analysis: Intravit–CRT: baseline vs. three months, *p* = 0.0008; baseline vs. six months, *p* < 0.0001. Intravit–TVFD: baseline vs. three months, *p* = 0.015; baseline vs. six months, *p* < 0.0001; three months vs. six months, *p* = 0.002.

**Table 2:** Overall treatment efficacy and therapy effects at follow-up measurements of the parameters.

Parameter	Overall Treatment Effect		Treatment Effect at the Two Follow-Up			
	Mean Difference (95%CI)	*p*	Three Months Mean Difference (95%CI)	*p*	Six Months Mean Difference (95%CI)	*p*
CRT (μm)	−24.24 (−38.87 to −9.81)	0.016	−19.74 (−34.24 to −5.25)	0.008	−28.89 (−43.87 to −13.90)	0.0003
Glycemia (mg/dL)	−6.81 (−13.39 to −0.23)	0.04	−6.02 (−12.74 to 0.70)	0.08	−7.63 (−14.22 to −1.04)	0.02
HbA1C (%)	−0.07 (−0.13 to −0.009)	0.02	−0.04 (−0.10 to 0.01)	0.11	−0.09 (−0.16 to −0.02)	0.017
Microaneurysm (count)	−0.07 (−0.26 to 0.13)	0.49	−0.04 (−0.11 to 0.02)	0.17	−0.09 (−0.17 to −0.01)	0.026
TVFD (%)	2.15 (1.40 to 2.91)	<0.0001	0.07 (0.03 to 0.11)	0.0005	0.10 (0.07 to 0.14)	<0.0001

Abbreviation: CRT: central retinal thickness; TVFD: total vascular flow density.

**Table 3:** Comparison of the parameters between the responders.

Parameter	Good	Poor	*p*
	Mean ± SD (95%CI)	Mean ± SD (95%CI)	
SCP (%)	19.54 ± 1.79 (18.70 to 20.38)	20.65 ± 2.41 (18.63 to 22.67)	0.19
DCP (%)	21.88 ± 2.27 (20.82 to 22.94)	16.54 ± 2.14 (14.75 to 18.33)	<0.0001
FAZ (mm^2^)	0.57 ± 0.18 (0.49 to 0.65)	0.82 ± 0.11 (0.73 to 0.91)	0.0013

Abbreviation: SCP: superficial capillary plexus; DCP: deep capillary plexus; FAZ: foveal avascular zone.
